# Hughes-Stovin syndrome—An important differential diagnosis in patients with suspected chronic thromboembolic pulmonary hypertension: A case report

**DOI:** 10.1007/s00508-023-02299-w

**Published:** 2023-11-15

**Authors:** Svitlana Pochepnia, Irene M. Lang, Ruxandra Iulia Milos, Sebastian Röhrich, Ahmed Ba-Ssalamah, Lucian Beer, Helmut Prosch

**Affiliations:** 1 Vienna General Hospital, Dept. of Biomedical Imaging and Image-guided Therapy, Medical University of Vienna, Währingergürtel 18–20, 1090 Vienna, Austria; 2 Dept. of Internal Medicine II, Division of Cardiology, Medical University of Vienna, Vienna, Austria

**Keywords:** Vasculitis, Hughes-Stovin syndrome, Pulmonary hypertension, Pulmonary arteries, Chronic thromboembolic pulmonary hypertension

## Abstract

Hughes-Stovin syndrome (HSS) is a rare vasculitis of unknown etiology. The disease is characterized by pronounced inflammation and damage to the vessel walls, with subsequent widespread vascular thrombosis and the formation of pulmonary artery aneurysms that can lead to fatal hemoptysis. This disorder can be mistaken for other conditions, such as chronic thromboembolic pulmonary disease (CTEPD) without or with pulmonary hypertension at rest (CTEPH).

We report the case of a 20-year-old female with HSS, which was misdiagnosed as CTEPH and subsequently treated with anticoagulants, which led to severe hemoptysis and eventually death of the patient. This case highlights the challenges of diagnosing HSS at early stages of the disease.

HSS should be considered in young patients with signs of large vessel vasculitis in combination with thrombotic occlusions of pulmonary arteries, with or without aneurysms of the pulmonary arteries, and particularly, if there are no risk factors for thromboembolic disease.

## Introduction

Hughes-Stovin syndrome (HSS) is a rare large vessel vasculitis characterized by recurrent thrombophlebitis, deep vein thrombosis, and an inflammation of the pulmonary arteries leading to in situ thrombosis and aneurysm formation [1]. Because the appearance of HSS in computed tomography (CT), as well as the clinical presentation, resemble chronic thromboembolic pulmonary disease (CTEPD) without or with pulmonary hypertension at rest (CTEPH), HSS can easily be misdiagnosed as CTEPD. The correct diagnosis of HSS is important because the treatment of HSS is very different from the treatment of CTEPD. We report the case of a 20-year-old female patient with HSS which was misdiagnosed as CTEPH and discuss the diagnostic challenges.

## Case presentation

A 20-year-old female patient with a presumptive diagnosis of CTEPH was referred to our hospital. The disease began 2.5 years earlier with flares of fever (up to 40 ^o^C), chills, and dyspnea. The patient was initially evaluated in another hospital, where the patient was diagnosed with CTEPH and put on anticoagulation. At presentation, the clinical examination was unremarkable. Apart from anemia, extensive laboratory investigations were normal.

**Fig. 1:**
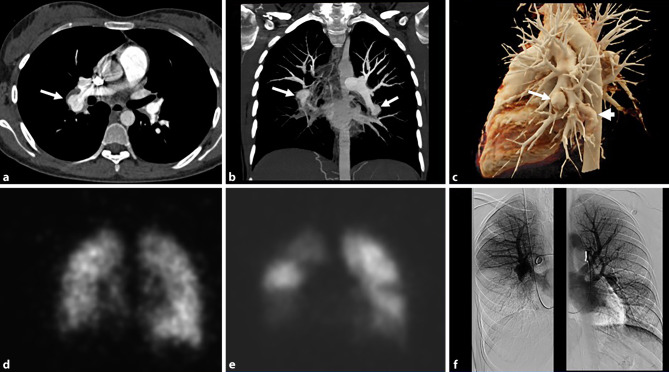
**a**–**c** CT pulmonary angiography: **a** axial reconstruction showing an aneurysm of the right lower lobar artery with wall-adherent thrombus (*arrow*). **b** Coronal maximum intensity projection (MIP) shows bilateral aneurysms or pseudoaneurysms of the right lower pulmonary artery and left lower lobe segmental pulmonary arteries (*arrows*). **c** 3D-VRT shows pulmonary aneurysm or pseudoaneurysm (*arrow*) and a varicose aneurysm on the left side (*arrowhead*). **d**–**e** Ventilation (**d**)-perfusion (**e**) scintigraphy. Coronal view scintigraphy shows extensive perfusion defects in both lower lung fields, the right upper lung field and left middle lung field. **f** Digital subtraction pulmonary angiography shows right pulmonary artery aneurysm with lower lobe perfusion defect and left segmental pulmonary artery aneurysm that corresponded to the results of perfusion scintigraphy

The CT showed a bilateral pulmonary artery aneurysm with wall-adherent thrombi and relatively well-defined walls of the aneurysms (Fig. 1a–c). Furthermore, thrombotic masses in the lumen of subsegmental and segmental arteries were observed. Ventilation-perfusion scintigraphy showed multiple perfusion defects in the lower lobe of both lungs, the apical right upper lobe and the lingula, compatible with CTEPH (Fig. 1d, e). On right heart catheterization, all pressures were normal. Pulmonary angiography showed massive aneurysm of the right lower lobe pulmonary artery with an occlusion of the pulmonary arteries and extensive segmental pulmonary artery aneurysms in the left lower lobe (Fig. 1f).

Ultrasound of the extremities revealed thrombophlebitis of the left cephalic vein of the forearm, a chronic occlusion of the right left cephalic vein of the forearm, and unremarkable veins of both lower extremities.

**Fig. 2:**
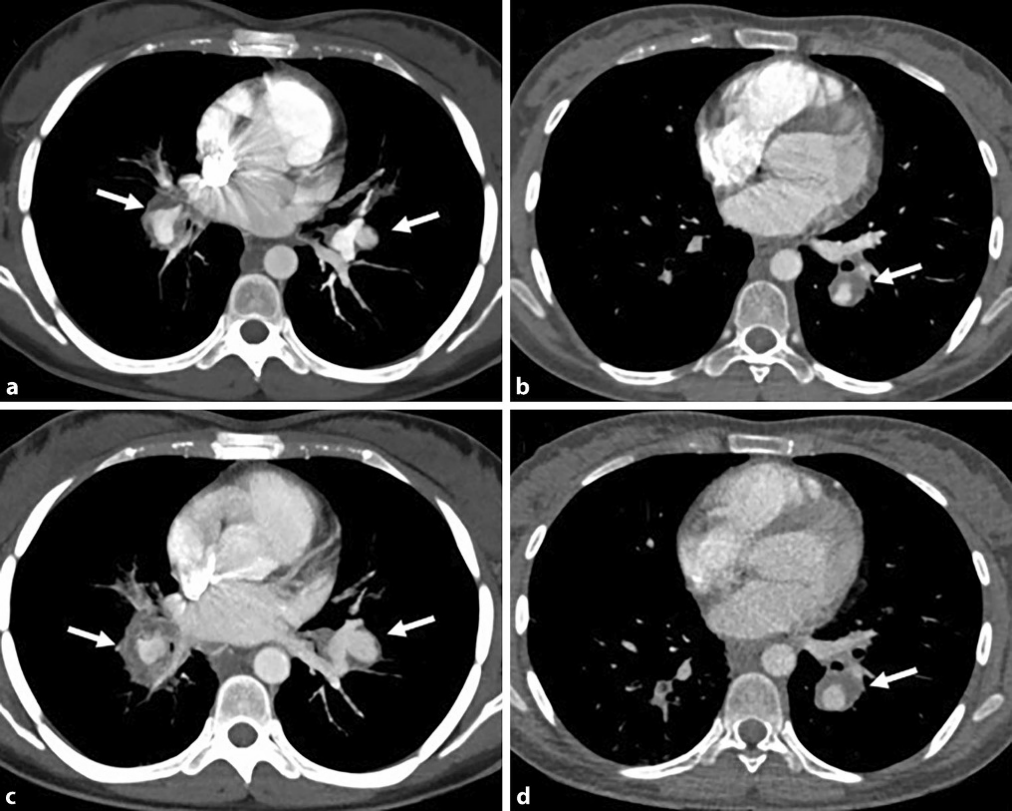
CT pulmonary angiography at presentation (**a** and **b**) and CT follow-up after 3 weeks (**c** and **d**) showing a progression of the pulmonary artery aneurysms on both sides (*arrows*) (on the right side, from 2 to 2.6 cm, on the left side, from 1.4 to 1.9 cm)

**Fig. 3:**
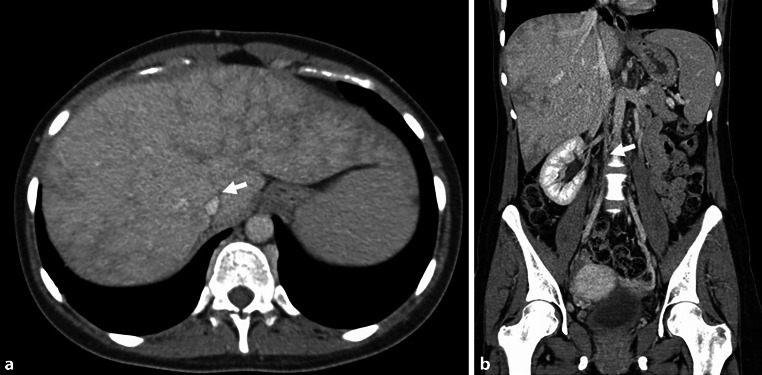
**a** The axial CT scan of the abdomen shows the Budd-Chiari syndrome with a thrombosis of the middle and left hepatic veins (*arrow*). **b** Coronal reformation of the CT shows a fibrotic occlusion of the inferior vena cava (*arrow*)

After one more severe episode of hemoptysis 3 weeks after initial presentation, the patient underwent another CT examination, which revealed progression of the diameter of the aneurysms of the main pulmonary arteries (Fig. 2a–d) and multiple patchy ground-glass opacites, compatible with a pulmonary hemorrhage. Abdominal CT showed a complete obliteration of the inferior vena cava due to thrombus and fibrotic changes over the entire length. The liver showed parenchymal perfusion abnormalities and thrombosis of the left and middle hepatic vein consistent with Budd-Chiari syndrome (Fig. 3).

Based on the combination of the imaging findings with the history of the patient and laboratory findings a Hughes-Stovin syndrome was diagnosed. The patient was discharged in good general condition on immunosuppressive therapy. The patient was re-admitted with massive hemoptysis 2 months after the first presentation, to which she succumbed.

## Discussion

HSS is a rare disease characterized by a large-vessel vasculitis, typically involving one or more pulmonary and bronchial arteries, leading to in situ thrombosis and pulmonary artery and/or bronchial artery aneurysm formation, as well as systemic venous thrombosis [1]. To date, there are no established diagnostic criteria, but a combination of pulmonary and/or bronchial artery aneurysms with in situ thrombosis and recurrent thrombophlebitis and/or deep vein thrombosis in different regions in the absence of other etiological factors and normal coagulation profile is considered the main feature of HSS [1]. HSS is considered a variant of Behçet’s disease, which shows oral and genital ulcers, and may be associated with thrombophlebitis, as well as pulmonary artery aneurysms [1].

The pathogenesis of aneurysm and thrombus formation in the pulmonary arteries in HSS remains controversial. The inflammation of vessel walls is most likely leading to the formation of aneurysms, which is also seen in Behçet disease, to which HSS shows several similarities [2]. It is still debated whether the thrombotic masses in the pulmonary arteries are formed in situ or are the result of emboli, or both. It has been primarily argued, however, that the thrombotic occlusions of the pulmonary arteries in HSS are due to an underlying arterial vasculitis rather than thromboembolism.

Especially in early stages of the disease, thrombotic occlusion of the pulmonary arteries without dilatation of the vessel lumen or formation of an aneurysm may be mistaken for pulmonary embolisms. The most important radiological evidence of vasculitis as the cause of thrombotic occlusion is contrast enhancement of the vessel wall adjacent to the thrombotic mass, which reflects inflammation of the vessel wall and is a typical finding for vasculitis due to HSS but is not observed in CTEPH [3, 4]. An additional approach to visualizing vessel wall inflammation in patients with suspected pulmonary arterial vasculitis is through 18F-fluorodeoxyglucose positron emission tomography computed tomography (FDG-PET-CT). The FDG-PET-CT has been demonstrated to yield positive results in anecdotal cases of Hughes-Stovin syndrome and Behcet’s disease [5, 6]. Consequently, it can be regarded as a supplementary modality for investigating patients with suspected HSS.

Because of the different treatment strategies, it is important to differentiate the nature of pulmonary occlusion: for patients with PE or CTEPD, the main treatment option is anticoagulation, while for patients with HSS, the main treatment option is immunosuppressive therapy, and anticoagulants should be administered with great caution [1, 7].
